# Characteristics of Received HIV Prevention Advocacy from Persons Living with HIV in Uganda, and Associations with HIV Testing and Condom Use Among Social Network Members

**DOI:** 10.1007/s10461-024-04347-6

**Published:** 2024-04-20

**Authors:** Glenn J. Wagner, Laura M. Bogart, Joseph K.B. Matovu, Stephen Okoboi, Violet Gwokyalya, David J. Klein, Susan Ninsiima, Harold D. Green

**Affiliations:** 1 RAND Corporation, 1776 Main Street, Santa Monica, CA 90407 USA; 2 Charles R. Drew University of Medicine and Science, Los Angeles, CA USA; 3 Makerere University School of Public Health, Kampala, Uganda; 4 Infectious Diseases Institute, Makerere University College of Health Sciences, Kampala, Uganda; 5 University of Indiana Bloomington School of Public Health, Bloomington, IN USA

**Keywords:** HIV, Uganda, Prevention, Advocacy, Peer, Social network, HIV testing, Condom use

## Abstract

Receiving peer advocacy has been shown to result in increased HIV protective behaviors, but little research has gone beyond assessment of the mere presence of advocacy to examine aspects of advocacy driving these effects. With baseline data from a controlled trial of an advocacy training intervention, we studied characteristics of HIV prevention advocacy received among 599 social network members of persons living with HIV in Uganda and the association of these characteristics with the social network members’ recent HIV testing (past six months) and consistent condom use, as well as perceived influence of advocacy on these behaviors. Participants reported on receipt of advocacy specific to HIV testing and condom use, as well as on measures of advocacy content, tone of delivery, support for autonomous regulation, and perceived influence on behavior. Receiving HIV testing advocacy and condom use advocacy were associated with recent HIV testing [65.2% vs. 51.4%; OR (95% CI) = 1.77 (1.11–2.84)], and consistent condom use with main sex partner [19.3% vs. 10.0%; OR (95% CI) = 2.16 (1.12–4.13)], respectively, compared to not receiving advocacy. Among those who received condom advocacy, perceived influence of the advocacy was positively correlated with consistent condom use, regardless of type of sex partner; support of autonomous regulation was a correlate of consistent condom use with casual sex partners, while judgmental advocacy was a correlate of consistent condom use with serodiscordant main partners. Among those who received testing advocacy, HIV testing in the past 6 months was positively correlated with receipt of direct support for getting tested. In multiple regression analysis, perceived influence of both HIV testing and condom use advocacy were positively correlated with advocacy that included access information and support of autonomous regulation; confrontational advocacy and judgmental advocacy were independent positive correlates of perceived influence of testing and condom use advocacy, respectively. These findings support associations that suggest potential benefits of peer advocacy from PLWH on HIV testing and condom use among their social network members, and indicate that advocacy content, tone of delivery, and support of autonomous regulation advocacy may play an important role in the success of advocacy.

## Introduction

In Uganda, HIV prevalence has stagnated at around 6% for over two decades, despite the widespread use of test-and-treat and combination biomedical approaches that have led to advancements in HIV testing, antiretroviral therapy (ART) coverage and viral suppression rates [1]. Some estimate the potential for rapid increases in HIV incidence, with contributing factors including high rates of condomless sex, individuals living with HIV not knowing their status, and access to pre-exposure prophylaxis (PrEP) being limited to key populations [2]. In 2022, the country began using a combination prevention approach that utilizes social network-based case finding to locate persons at high risk for HIV, scale up of HIV self-testing, assisted partner notification, and optimization of provider-initiated testing and counselling [3]. Nonetheless, further development of innovative strategies that are not resource intensive, and that can reach a large number of people efficiently, is needed to make greater inroads on HIV prevention.

Drawing from theories of social diffusion [4] and social influence [5], and evidence of peer advocacy’s beneficial effects on HIV protective behaviors [6–8], persons living with HIV (PLWH) have the potential to act as powerful change agents for HIV prevention by advocating for HIV protective behaviors within their social networks and communities. In doing so, PLWH become part of the solution to battling HIV, which can help counter the stigmatizing view that they are the problem. PLWH can be credible and influential when conveying prevention messages to family and friends, given their close relations with these individuals and their ability to exemplify the benefits of HIV testing and care on health [9]. PLWH in Uganda typically have dense, interconnected networks [10], and have disclosed to many people in their networks [11], which suggests that transfer of HIV prevention messages and knowledge through one’s network can be very efficient.

Our prior research in Uganda has shown that essentially all PLWH actively engage in some form of HIV prevention advocacy by encouraging at least some friends and family to seek HIV testing and care and to reduce HIV risk behaviors [8, 12, 13]. Furthermore, social network members who receive consistent condom use advocacy over time from PLWH are more likely to report consistent condom use (70%) than social network members who receive less advocacy (36%), highlighting the potential value of ongoing peer advocacy for promoting HIV risk reduction [14]. However, advocacy in these studies was assessed only in terms of the presence of advocacy, not the nature or content of the advocacy (e.g., provision of access information, direct support), perceived tone of delivery (e.g., caring, non-judgmental), support of autonomous regulation (i.e., helping the target of advocacy make their own decision about behavior change), or perceived influence on behavior from the perspective of the person receiving the advocacy.

To address each of these gaps in the HIV peer advocacy literature, we analyzed survey data from a large sample of social network members of PLWH in Uganda to assess multiple characteristics of prevention advocacy received from PLWH as part of their natural relationship (i.e., before the PLWH received an advocacy training intervention), and any associations of those aspects with HIV protective behaviors (i.e., HIV testing, consistent condom use).

## Methods

### Study Design

We conducted a cross-sectional correlational analysis with baseline data from an ongoing randomized controlled trial of a group intervention that empowers PLWH to engage in HIV prevention advocacy with members of their social network. The study protocol was approved by the Infectious Diseases Institute Research Ethics Committee and the Human Subjects Protection Committee at the RAND Corporation and registered with the Uganda National Council of Science and Technology. PLWH are enrolled in the study as index participants, and each index participant refers members of their social network (also called “alters”) to enroll. Further details of the study intervention and protocol are available in a prior publication [15]. The analysis reported in this paper is based solely on baseline data from alter participants.

### Participants

Recruitment took place between January 2022 and February 2023 in Kampala, Uganda at The Infectious Diseases Institute (IDI), which provides outpatient HIV care to over 8,000 PLWH. Eligibility criteria for PLWH included: (1) age ≥ 18 years, (2) in HIV care for at least one year (because they are more likely to be medically stable, adjusted to their HIV diagnosis, and have disclosed to several people, and thus more likely to be ready to engage in advocacy), (3) speak fluent Luganda, (4) no signs of significant cognitive impairment (based on interviewer observation), and (5) does not have a partner/spouse or household member already enrolled in the study as an index participant. A sample of 210 PLWH index participants was enrolled.

To recruit alters, during the baseline interview of the index participant we elicited the names of up to 20 alters in their social network who they have the most contact with and asked the participant to identify the alters who know the index participant’s HIV serostatus. At the end of the interview, the index participant was asked to select from the list of alters who know their serostatus up to seven alters whom they would be willing to refer to the study. If they selected more than four alters, we randomly selected four to target for recruitment (to limit selection bias). The index participant was asked to call each selected alter after their baseline interview and describe the study in the presence of the coordinator, who could then immediately schedule a study visit for the alter. If any of the alters refused to participate or could not be reached, the coordinator randomly selected an additional alter from the list of those referred by the index participant until four had agreed and enrolled; if only four were referred, we asked the index participant to refer additional alters, if possible. Midway through the study, we allowed index participants to refer more than four alters for recruitment to reach our enrollment goal for alter participants.

### Measures

The assessment was interviewer-administered using Network Canvas software [16] and conducted in Luganda. All measures were developed by the study team and had been translated from English to Luganda using a standard translation/backtranslation methodology from a prior study [8]. Participants received 30,000–70,000 Uganda shillings (~$8–20 USD), depending on the distance traveled, after completing the assessment to cover transportation costs.

**HIV protective behaviors.*Condom use*** in the past six months with main and casual sex partners—in separate questions—was assessed. Participants who reported having a main sex partner and having sexual intercourse with that partner in the past six months were asked how frequently they used condoms during sexual intercourse with their main partner; response options ranged from 1 ‘never’ to 5 ‘always.’ This condom use item was also asked regarding sexual intercourse with casual sex partners in the past six months, if present. For condom use with both main and casual partners, a binary variable was created to represent consistent condom use, defined as always using condoms in the past six months. To assess ***HIV testing*** behavior the participant was first asked if they had ever tested for HIV; those who had tested were then asked to report when their last HIV test was done and the result of that test. A binary variable was created to represent recent HIV testing, defined as the most recent test being within the past six months. Participants who reported being HIV-positive were asked if they were ***receiving HIV care*** and if they were ***on ART*** in separate items. Participants who did not report being HIV-positive were asked about ***use of PrEP*** (in their lifetime and currently).

**Receipt of HIV prevention advocacy**. Receipt of advocacy in the past three months was assessed by asking the alter participant in separate items if their index participant had talked with them about (1) HIV testing (if alter was not HIV-positive, or had tested HIV-positive less than three months ago), (2) condom use (all alters), (3) use of PrEP (if alter was not HIV-positive), and—if alter was HIV-positive—(4) linkage to HIV care and (5) use of ART. For each of these five areas of advocacy, if such a conversation with their index participant had taken place, the following series of follow-up questions were asked to further assess aspects of the advocacy received from the index participant:

***Advocacy content*** was assessed by asking alter participants if the conversation included the following actions to promote the alters use of the behavior: (1) encouragement, (2) provision of information related to access (e.g., where to get an HIV test), and (3) provision of direct support (e.g., accompanied alter to the clinic); the response option for each question was 0 ‘no’ or 1 ‘yes’.

***Tone of advocacy delivery*** was assessed by asking the alter participant if the index participant (1) showed caring, (2) was confrontational, or (3) was judgmental, during the conversation, in separate items; response options were 1 ‘not at all’, 2 ‘a little’, 3 ‘somewhat’, and 4 ‘very much’.

***Support for autonomous regulation*** (i.e., supporting someone to make their own decision about use of a behavior) was assessed by asking the alter participant to indicate which of the following two statements was most true about the conversation: the index participant “told you what to do” or “helped you figure out what you needed to do” regarding the targeted HIV protective behavior. Autonomous regulation was present when the alter indicated that the index helped them figure out what they needed to do.

***Perceived influence*** of the conversation on use of the targeted behavior was assessed by asking the alter participant to rate “How much do you think the conversation influenced your [specific target behavior]?” on a 10-point scale with anchors being 1 ‘not at all influential’, 5 ‘somewhat influential’ and 10 ‘very influential’.

**Socio-demographic characteristics** included age (in years), gender (male, female), highest level of education, nature of relationship with index participant, and relationship characteristics (presence of a spouse, romantic partner, or main sex partner; HIV status of that partner).

### Data Analysis

Descriptive statistics were used to examine background characteristics of the sample. Bivariate logistic regression models were used to compare groups defined by binary measures of HIV protective behavior use (e.g., HIV testing in past 6 months, consistent condom use), while linear regression models were used to compare groups defined by continuous measures of advocacy (e.g., rating of influence). Note that for the measure of receipt of HIV testing advocacy, about one-quarter of the sample was not administered this measure due to an erroneous skip pattern that was corrected midway through recruitment. If more than one variable was at least marginally (*p* < .10) associated with the outcome behavior in bivariate analysis, we conducted multiple regression analysis to further assess independent correlates of the outcome behavior; these models included socio-demographic covariates (age, gender, any secondary education, nature of relationship with index participant). Multiple regression analysis related to consistent condom use was not considered for the subgroups of those with a serodiscordant main partner (*n* = 25) and those with casual sex partners (*n* = 51), as these groups were too small for a valid analysis.

For all bivariate and multiple regression analyses, effects of clustering (i.e., potential correlation of responses from alters from the same referring index participant) were accounted for by adjusting standard errors using regression software designed for complex data (SAS v9.4, Proc Surveylogistic and Proc Surveyreg). For the advocacy measures related to presence of encouragement and level of caring, some subgroups had no variance (i.e., all participants gave the same response). In these few instances, we used the Firth correction [17], although this method cannot accommodate adjusting standard errors for clustering.

## Results

### Sample Characteristics

A sample of 599 alters (recruited by 193 of the 210 enrolled PLWH index participants) consented to participate in the study and completed the baseline interview. The mean number of alters recruited by the 193 index participants was 3.1 (SD = 1.3; range: 1–7). Table 1 shows characteristics of enrolled alter participants. Most alter participants were female (66.3%) and average age was 37.4 years (SD = 12.4; range: 18–81). Three-quarters (*n* = 459; 76.6%) had a main sex partner, including 429 who were in a committed romantic relationship; for 94 (15.7% of total sample) participants, their main sex partner was reported to have an HIV status that was discordant from their own (i.e., one member of the dyad was HIV-positive and the other was not). Nearly all had tested for HIV in their lifetime (*n* = 578; 96.5%), of whom just over a third (*n* = 207; 35.8%) reported being HIV-positive. Nearly all who reported being HIV-positive were in HIV care (*n* = 204; 98.7%) and on ART (*n* = 203; 98.3%). Among those who reported not being HIV-positive, only 11 reported ever using PrEP, including 6 who were current users.

Due to the very high rates of HIV care and ART use among HIV-positive alters and the very low rate of PrEP use among HIV-negative alters we concentrated our analysis on the prevalence and correlates of recent HIV testing and condom use advocacy received by the alter participants.

### Relationship Between Received Condom Use Advocacy and Consistent Condom Use with Main Sex Partner

Among the 459 participants with a main sex partner, 87 of these partnerships involved both the alter and their partner being HIV-positive and were thus excluded from this analysis due to lack of risk of HIV transmission. Of the remaining 372 alters with a main sex partner, 318 (85.5%) reported having sexual intercourse with this partner at least once in the past six months. Of these 318, 40 (12.6%) reported always using condoms during sex with their main sex partner in the past six months. Those who received condom advocacy (*n* = 88) in the past three months from their index participant had more than twice the odds of always using condoms [19.3% vs. 10.0%; OR (95% CI) = 2.16 (1.12–4.13)], compared to the 230 who did not receive condom advocacy. Among those who received any condom use advocacy, greater perceived influence of advocacy was associated with consistent condom use [OR (95% CI) = 1.35 (1.00-1.84)](see Table 2).

This analysis was repeated among those with a main sex partner whose HIV status was discordant from their own and who reported having sexual intercourse with this partner at least once in the past six months (*n* = 79). Those who received condom advocacy (*n* = 25) did not differ in their odds of always using condoms [32.0% vs. 24.1%; OR (95% CI) = 1.48 (0.54–4.10)], compared to the 54 who did not receive condom advocacy. Among those who did receive advocacy, advocacy that was perceived as more influential [OR (95% CI) = 1.97 (1.04–3.73)], and more judgmental [OR (95% CI) = 2.33 (1.50–3.60)], were each associated with consistent condom use.

### Associations of Condom Use Advocacy with Casual Sex Partner Condom Use

One hundred and fourteen participants had casual sex partners in the six months prior to baseline, of whom 39 (34.2%) reported always using condoms with these partners in this time frame. Those who received condom advocacy (*n* = 51) did not differ in their odds of always using condoms [37.3% vs. 31.7%; OR (95% CI) = 1.28 (0.58–2.81)], compared to the 63 who did not receive condom advocacy. Among those who did receive advocacy, advocacy that was perceived as more influential [OR (95% CI) = 1.40 (1.04–1.88)], and supportive of autonomous regulation [OR (95% CI) = 4.25 (1.09–16.55)], were each associated with consistent condom use (see Table 2).

### Recent HIV Testing and the Relationship to Receipt of HIV Testing Advocacy

HIV testing in the past six months was relevant for 398 participants: 392 who did not report being HIV-positive and 6 participants who reported testing HIV-positive less than 6 months ago. Of these 398, 184 (46.2%) reported being tested for HIV in the past six months. Of the 398 participants, data related to receipt of testing advocacy was administered to 304 participants. Among these 304, 178 (58.6%) had tested for HIV in the past six months; those who received HIV testing advocacy in the prior 3 months (*n* = 158) were more likely to have been tested for HIV in the last 6 months [65.2% vs. 51.4%; OR (95% CI) = 1.77 (1.11–2.84)], compared to those who did not receive testing advocacy (*n* = 146). Among those who had received testing advocacy, advocacy that included direct support for accessing testing was associated with being tested for HIV in the past 6 months [OR (95% CI) = 2.65 (1.10–6.36)](see Table 3).

### Correlates of Perceived Influence of Advocacy on Condom Use and HIV Testing

Among participants who reported having sexual intercourse in the past 6 months either with a main partner (excluding alters living with HIV who had main partners who were also living with HIV) or casual sex partner, bivariate linear regression models showed that the perceived level of influence of condom use advocacy was positively correlated with each form of advocacy content measured (encouragement, access information, direct support), as well as with advocacy that included support for autonomous regulation and with advocacy that was delivered with higher levels of caring, confrontation or judgment (see Table 4). In multiple linear regression analysis, advocacy that was perceived to be more judgmental [beta (SE) = 0.78 (0.34); *p* = .023] was independently associated with greater perceived influence of advocacy on condom use behavior (see Table 4).

In bivariate analysis, perceived influence of HIV testing advocacy was positively correlated with advocacy that included access information, supported autonomous regulation, and that was perceived as confrontational and judgmental (see Table 4); advocacy that included direct support for access to condoms was a marginal correlate. In multiple linear regression analysis, inclusion of access information [beta (SE) = 1.15 (0.28); *p* < .0001], support for autonomous regulation [beta (SE) = 0.97 (0.29); *p* = .001], and greater use of confrontation [beta (SE) = 0.34 (0.14); *p* = .02] were each independently associated with greater perceived influence of advocacy on HIV testing (see Table 4).

## Discussion

In this analysis of the HIV prevention advocacy that occurs between PLWH in Uganda and their social network members (i.e., alters) as part of their natural relationship (i.e., before the PLWH received the advocacy training intervention), many alters reported receiving advocacy from the PLWH (who recruited them into the study) to engage in HIV protective behaviors. Receipt of HIV testing advocacy and receipt of condom use advocacy were associated with a higher likelihood of recent HIV testing and always using condoms with main sex partners, respectively. Among those who received advocacy, several aspects of advocacy—including nature of content, tone of delivery, promotion of autonomous regulation and perceived influence on behavior—were associated with consistent condom use or recent HIV testing by the alter.

Aside from the mere presence of advocacy, this analysis examined several characteristics of advocacy and their relationship to the desired target behavior by the alter. Analysis of the specific content of advocacy (i.e., encouragement, access information, direct support) revealed that provision of direct support for accessing the target HIV prevention resource (i.e., HIV testing, condoms) was only a significant correlate of alter HIV testing. This highlights the need for this more tangible source of support in the context of promoting HIV testing; perhaps there is greater knowledge of how and where to access condoms among the population as a whole, relative to knowledge of where to access HIV testing, resulting in the latter being more useful. Regarding the tone of how advocacy was delivered, perception of the index participant being more judgmental during condom use advocacy was associated with consistent condom use during sex with main partners who were serodiscordant. There were no other significant associations between tone of advocacy and alter use of HIV testing or condom use. Our data also showed that condom use advocacy that supported autonomous regulation (i.e., helping the person make their own decision about condom use) was associated with consistent condom use with casual sex partners, which aligns with research that shows the importance of autonomous regulation for enhancing motivation to change behavior [18, 19].

Alter’s perception of the influential of condom use advocacy on their condom use behavior was positively correlated with consistent condom use with each type of sex partner (i.e., main, main sero-discordant, casual). Most measured aspects of advocacy were correlated with alter perception of the influence of advocacy on their HIV testing and condom use behavior in bivariate analysis. Inclusion of encouragement, access information, direct support, support for autonomous regulation, and higher ratings of caring, confrontation and judgment were all positively correlated with higher perceived influence of HIV testing and/or condom use advocacy. In multiple regression analysis, advocacy that included access information and was supportive of autonomous regulation were each independently correlated (at least marginally) with perceived influence of both HIV testing and condom use advocacy. These findings highlight the value of information on how to access HIV testing and condoms and respect for a person being able to make their own decision about whether to use condoms, consistent with principles of motivational enhancement [18, 19]. In addition, advocacy perceived as more confrontational and advocacy perceived as more judgmental were independently correlated with perceived influence of HIV testing and condom use advocacy, respectively.

The benefits of advocacy using confrontation and/or judgmentalism (on alter condom use behavior and perceived influence of both testing and condom advocacy) were unexpected as they seem counter to the principles of motivational enhancement [17, 18]. One might expect that the use of confrontation and judgment may make the target of advocacy more defensive and less open to adopt the target behavior. However, our findings seem to suggest that strategies that more directly confront people with health sabotaging aspects of their behavior may be more apt to lead to behavior change. This may be particularly plausible if the person confronting and judging is trusted and respected by the person receiving the advocacy, and when autonomous regulation remains respected [20]. Although purely conjecture, the use of confrontation and/or judgment may be considered normative, as well as influential on social norms, in the context of communication about the use of HIV protective behaviors. This may contribute to a positive impact on both behavior and perception of positive influence. These findings have implications for advocacy training and the need to have a more nuanced approach for when to appropriately use confrontation and judgment, while still conveying care and support for autonomous regulation. However, given the counterintuitive findings and the small sample size within the subgroups involved in this part of the analysis, further research is needed to assess whether these findings are replicated.

Limitations of these analyses include the cross-sectional nature of the data, which precludes making any causal inferences between advocacy received and behavior targeted by advocacy. It is plausible that the associations between advocacy and behavior are bidirectional. Conversations about HIV testing and condom use could be prompted by a perception of the presence of HIV risk and the need to engage in the behaviors, or, conversely, as a reaction to or reflection on the already existing behavior. Other limitations included low sample size for some subgroups (e.g., alters in sexually active serodiscordant relationships), and skewed distributions for some measures (e.g., presence of encouragement, and caring tone of delivery), resulting in reduced statistical power. Furthermore, our measures of advocacy content, tone of delivery and influence are vulnerable to bias related to recall memory, participant interpretation of the construct (e.g., the wording of the statement intended to reflect perceived support for autonomous regulation does not provide a clear representation of the level of influence that the index participant tried to exert, which could vary how the respondents interpreted the statement), and single item measurement. Lastly, future research should assess the frequency of advocacy conversations, as advocacy that takes place over time may differ in its influence on behavior beyond the mere presence of any advocacy.

In conclusion, these findings suggest that receipt of HIV testing and condom use advocacy from PLWH are associated with the use of these behaviors among their social network members, and that aspects of the content, tone of delivery, and support of autonomous regulation may be important for advocacy to impact these HIV protective behaviors. Advocacy in the form of direct support for access appears to be important for promoting HIV testing, while advocacy that is supportive of autonomous regulation appears to be important for promoting condom use. Further research is needed to better understand the mechanisms by which specific aspects of advocacy may drive its impact on target behaviors, including content (e.g., differentiating specific forms of information and direct support), tone of delivery (e.g., how do confrontation and judgment affect change?), and the nature of support for autonomous regulation (e.g., is some level of index influence or direct advice needed to affect change?). Overall, our findings can inform advocacy training interventions designed to improve engagement in HIV protective behaviors among social network members of PLWH.

**Table 1:** Sample characteristics at baseline (*n* = 599)

	Mean (SD)/%
**Sociodemographics**	
Age	37.4 (12.4)
Any secondary education	393/598 (65.7%)
Male	202 (33.7%)
Salaried employment	111/597 (18.5%)
Had a main sex partner	459 (76.6%)
Had a main sex partner whose HIV status is serodiscordant	94 (15.7%)
Had casual sex partners in past 6 months	117 (19.5%)
Relationship to index participant:^1^	
Family	341 (57.3%)
Friend	194 (32.6%)
Sex/romantic partner	60 (10.1%)
**HIV disease**	
Ever HIV tested	578 (96.5%)
Among those who have tested (n = 578): Self-reported living with HIV	207 (35.8%)
Among those living with HIV (n = 207): In HIV care	204 (98.6%)
Among those living with HIV (n = 207): on ART	203 (98.1%)
Among those not reporting to be living with HIV (n = 371): Ever on pre-exposure prophylaxis (PrEP)	11 (3.0%)
**Received HIV prevention advocacy from index (past 3 months)**	
Had discussions with index about HIV testing	158 (51.8%)
Among those who reported such discussions:	
Index provided encouragement	157 (99.4%)
Index provided information	112 (70.9%)
Index provided direct support	40 (25.3%)
Perceived usefulness of the discussion	3.85 (0.51)
Index was caring during the discussion	3.95 (0.27)
Index was confrontational during the discussion	1.22 (0.72)
Index was judgmental during the discussion	1.15 (0.59)
Index supported autonomous regulation	91 (57.6%)
Perceived influence of the discussion on testing behavior	7.69 (1.89)
Had discussions with index about condom use	106 (29.2%)
Among those who reported such discussions:	
Index provided encouragement	100 (94.3%)
Index provided information	64 (60.4%)
Index provided direct support	49 (46.2%)
Perceived usefulness of the discussion	3.70 (0.77)
Index was caring during the discussion	3.87 (0.48)
Index was confrontational during the discussion	1.36 (0.94)
Index was judgmental during the discussion	1.18 (0.66)
Index supported autonomous regulation	64 (60.4%)
Perceived influence of the discussion on condom use behavior	6.91 (2.37)

SD = standard deviation; ART = antiretroviral therapy

^1^*N* = 595; 4 had missing data

**Table 2:** Bivariate logistic regression models examining correlates of condom advocacy characteristics and consistent condom use in past 6 months by partner type, among alters who received condom advocacy from their index participant

Advocacy characteristics	Main sex partner^1^			Main sex partner is sero-discordant			Casual sex partners		
	Not always use condoms (*n* = 71)	Always use condoms (*n* = 17)	OR (95% CI)	Not always use condoms (*n* = 17)	Always use condoms (*n* = 8)	OR (95% CI)	Not always use condoms (*n* = 32)	Always use condoms (*n* = 19)	OR (95% CI)
Encouragement	66 (93.0%)	17 (100%)	2.90 (0.12-72.24)^2^	15 (88.2%)	8 (100%)	2.74 (0.06-124.4)^2^	31 (96.9%)	19 (100%)	1.86 (0.02-179.06)^2^
Access information	45 (63.4%)	10 (58.8%)	0.83 (0.26 − 2.60)	8 (47.1%)	4 (50%)	1.13 (0.18-7.01)	19 (59.4%)	13 (68.4%)	1.48 (0.36-6.05)
Direct support	33 (46.5%)	7 (41.2%)	0.81 (0.26-2.49)	9 (52.9%)	3 (37.5%)	0.53 (0.09-3.29)	15 (46.9%)	11 (57.9%)	1.56 (0.44 − 5.50)
Autonomous regulation	42 (59.2%)	11 (64.7%)	1.27 (0.40-4.01)	12 (70.6%)	7 (87.5%)	2.92 (0.22-37.95)	**15 (46.9%)**	**15 (78.9%)**	**4.25 (1.09–16.55)**
Caring	3.86 (0.52)	4.00 (0.00)	1.71 (0.31-9.31)^2^	3.59 (0.87)	4.00 (0)	2.10 (0.35-12.76)^2^	3.69 (0.78)	3.95 (0.23)	2.74 (0.82-9.13)
Confrontational	1.28 (0.85)	1.59 (1.18)	1.36 (0.91-2.03)	1.18 (0.73)	1.50 (1.07)	1.54 (0.93-2.57)	1.38 (1.01)	1.63 (1.26)	1.23 (0.73-2.07)
Judgmental	1.18 (0.64)	1.18 (0.73)	0.98 (0.41-2.38)	**1.06 (0.24)**	**1.38 (1.06)**	**2.33 (1.50–3.60)**	1.13 (0.55)	1.47 (1.12)	1.68 (0.80-3.54)
Influenced behavior	6.77 (2.31)	8.18 (2.04)	1.35 (1.00-1.84)	**5.94 (2.16)**	**8.75 (1.83)**	**1.97 (1.04–3.73)**	**6.17 (2.60)**	**7.79 (1.69)**	**1.40 (1.04–1.88)**

OR = odds ratio; CI = confidence interval; bold = *p* < .05

^1^ Excluded alters who were living with HIV and their main partner was also living with HIV

^2^ At least one cell in this comparison was zero; therefore, the Firth correction was applied

**Table 3:** Bivariate logistic regression models examining correlates of advocacy characteristics and alter HIV testing in past 6 months, among alters who received testing advocacy from their index participant (*n* = 398,^1^ but data only available for 304)

Advocacy characteristics	Not tested (*n* = 126)	HIV tested (*n* = 178)	OR (95% CI)
Encouragement^2^	55 (100%)	102 (99.0%)	0.61 (0.01-57.65)
Access information	38 (69.1%)	74 (71.8%)	1.14 (0.53-2.48)
Direct support	**8 (14.5%)**	**32 (31.1%)**	**2.65 (1.10–6.36)**
Autonomous regulation	32 (59.3%)	59 (57.3%)	0.92 (0.45-1.89)
Caring	3.93 (0.33)	3.96 (0.24)	1.54 (0.61-3.92)
Confrontational	1.29 (0.81)	1.17 (0.66)	0.81 (0.48-1.35)
Judgmental	1.22 (0.74)	1.12 (0.49)	0.76 (0.44 − 1.30)
Influence on behavior	7.51 (2.29)	7.78 (1.64)	1.08 (0.89-1.31)

^1^ Subgroup of participants who did not report to be HIV-positive or who tested HIV-positive within past three months; data were only available for 304 of these participants because of a faulty skip pattern that was not rectified until midway through the study

^2^ At least one cell in this comparison was zero; therefore, the Firth correction was applied

OR = odds ratio; CI = confidence interval; bold = *p* < .05

**Table 4:** Bivariate and multiple linear regression models of the relationships between perceived influence of received advocacy for HIV testing and condom use and characteristics of advocacy, among those who received HIV testing and condom use advocacy

Advocacy characteristics	Influence of HIV testing advocacy (*n* = 156)^1^		Influence of condom advocacy (*n* = 104)^2^	
	Bivariate linear regression beta (SE); p	Multiple linear regression^4^ beta (SE); p	Bivariate linear regression beta (SE); p	Multiple linear regression^4^ beta (SE); p
Included encouragement	---^3^	---	2.38 (0.79); 0.004	2.08 (1.15); 0.074
Included information on how to access	1.40 (0.34); <0.0001	1.15 (0.28); <0.0001	1.57 (0.50); 0.002	0.92 (0.51); 0.077
Included direct support to access	0.62 (0.33); 0.06	0.27 (0.32); 0.41	0.97 (0.47); 0.040	0.59 (0.47); 0.219
Promoted autonomous regulation	1.13 (0.30); 0.0003	0.97 (0.29); 0.001	1.19 (0.47); 0.014	0.71 (0.44); 0.114
Demonstrated caring	0.99 (0.64); 0.12	---	1.07 (0.34); 0.002	0.79 (0.44); 0.079
Confrontational	0.50 (0.14); 0.001	0.34 (0.14); 0.02	0.54 (0.25); 0.038	0.05 (0.33); 0.889
Judgmental	0.47 (0.17); 0.007	0.16 (0.21); 0.45	0.80 (0.22); 0.001	0.78 (0.34); 0.023

SE = standard error

^1^ Subgroup of participants who did not report to be HIV-positive or who tested HIV-positive within past three months, and who reported receiving testing advocacy from their index participant in the past 3 months (*n* = 158 minus two with missing data for perceived influence—hence *n* = 156 for the analysis)

^2^ Subgroup of participants who reported having sexual intercourse with a main partner (who was not concordant with the alter in being HIV-positive) or casual sex partner in the past 6 months, and who reported receiving condom advocacy from their index participant in the past 3 months (*n* = 106 minus two with missing data for perceived influence; hence *n* = 104 for the analysis)

^3^ Regression model was not run, because all but one participant responded “yes” on this measure

^4^ Multiple regression analysis included variables that were at least marginally correlated with influence in the bivariate models; covariates included age, gender, any secondary education, and nature of relationship to index participant

## Data Availability

De-identified dataset and statistical code are available to researchers upon submission of the proposal and review by the study team.
